# Early First Use of Psychoactive Substances: Long-Term Social and Professional Consequences in Adulthood

**DOI:** 10.1192/j.eurpsy.2026.10897

**Published:** 2026-07-31

**Authors:** I. Bouamoud, Z. Bencharfa, F. El omari

**Affiliations:** 1 Pedopyschiatry; 2 Addictology , Arrazi hospital, Salé , Morocco

## Abstract

**Introduction:**

Early use of psychoactive substances constitutes a major factor of social vulnerability. It often precedes school, professional, and family disruptions, fostering isolation, precariousness, and exclusion from healthcare systems. This phenomenon, still underexplored in the Moroccan context, raises concerns for prevention and support programs.

**Objectives:**

To analyze the social and professional consequences of early substance use among young adults hospitalized for substance use disorder, and to identify potential levers for collective action.

**Methods:**

A retrospective, descriptive, and analytical study conducted among patients hospitalized in the addiction unit of the Arrazi University Hospital. Data were collected using a structured questionnaire assessing age of first use, consumption profile (polysubstance use, relapses), and social impacts (job loss, isolation, precariousness, family breakdown, legal history).

**Results:**

Subjects who started early present a higher risk of social disaffiliation. The accumulation of comorbidities, relapses, and economic vulnerabilities reinforces social exclusion. The influence of family context and living environment is particularly significant.

**Conclusions:**

Early addictions must be recognized as a major societal challenge. Collective responses, combining integrated prevention, community support, and reintegration assistance, are necessary to break the cycle of exclusion.

**Disclosure of Interest:**

None Declared

